# 

**Published:** 1974-07

**Authors:** 


Bristol Medico-Chirurgical Journal Vol. 89 (iii) No. 331

### Contents

Editorial ... ... ... ... ... ... ... ... ... ... 23

The History of Medicine in Bristol and of the Bristol Medico-Chirurgical Society ... 25
C. Bruce Perry

Medical Journalism in the West of England 1883-1970 ... ... ... ... 31
N. J. Brown

The History of Psychiatry in Bristol ... ... ... ... ... ... 45

R. F. Barbour

Bristol Medico-Chirurgical Society ... ... ... ... ... ... 55, 58

The Centenary Dinner ... ... ... ... ... ... ... ... 56

University of Bristol ... ... ... ... ... ... ... ... 58

F. Bailey & Son Ltd., Dursley, Glos.

